# Protein Profile Changes during Porcine Oocyte Aging and Effects of Caffeine on Protein Expression Patterns

**DOI:** 10.1371/journal.pone.0028996

**Published:** 2011-12-16

**Authors:** Guang-Jian Jiang, Ke Wang, De-Qiang Miao, Lei Guo, Yi Hou, Heide Schatten, Qing-Yuan Sun

**Affiliations:** 1 State Key Laboratory of Reproductive Biology, Institute of Zoology, Chinese Academy of Sciences, Beijing, People's Republic of China; 2 College of Life Science, He-Bei Union University, Tangshan, People's Republic of China; 3 Department of Veterinary Pathobiology, University of Missouri, Columbia, Missouri, United States of America; National Cancer Institute, United States of America

## Abstract

It has been shown that oocyte aging critically affects reproduction and development. By using proteomic tools, in the present study, changes in protein profiles during porcine oocyte aging and effects of caffeine on oocyte aging were investigated. By comparing control MII oocytes with aging MII oocytes, we identified 23 proteins that were up-regulated and 3 proteins that were down-regulated during the aging process. In caffeine-treated oocytes, 6 proteins were identified as up-regulated and 12 proteins were identified as down-regulated. A total of 38 differentially expressed proteins grouped into 5 regulation patterns were determined to relate to the aging and anti-aging process. By using the Gene Ontology system, we found that numerous functional gene products involved in metabolism, stress response, reactive oxygen species and cell cycle regulation were differentially expressed during the oocyte aging process, and most of these proteins are for the first time reported in our study, including 2 novel proteins. In addition, several proteins were found to be modified during oocyte aging. These data contribute new information that may be useful for future research on cellular aging and for improvement of oocyte quality.

## Introduction

The quality of an oocyte plays a critical role in embryonic developmental potential after fertilization [1]. In most mammalian species, oocytes are arrested at the metaphase stage of second meiosis (MII) before fertilization takes place, but oocytes will undergo an aging process both *in vivo* and *in vitro* if fertilization does not occur in time [2], [3]. It has been well established that aged oocytes display many functional changes, including decreased fertilization rates [4], polyspermy [5], chromosomal anomalies [6], and abnormal development of embryos [7]. Therefore, research on oocyte aging is important for reproductive health.

It is thought that the oocyte aging process is related to changes in concentration of calcium ions [8], reactive oxygen species [9], activity of M-phase promoting factor (MPF) and mitogen-activated protein kinase (MAPK) [10]. Interestingly, it has been reported that oocyte aging is reversible through controlling MPF activity [11], [12]. Kikuchi *et al*. reported that oocyte aging was prevented by treating oocytes with caffeine [12]. Caffeine induced dephosphorylation of the catalytic subunit of MPF, p34cdc2, to elevate the activity of MPF. This has also been found in cultured mammalian cells [13] and *Xenopus laevis* oocytes [14]. However, the exact biological process induced by caffeine is still not clear due to limitations in technology.

With proteomic tools, differential proteins can be detected systematically. Here, we explored protein profile changes during porcine oocyte aging and the effects of caffeine on protein changes with two-dimensional Difference Gel Electrophoresis (2D DIGE) combined with Matrix-Assisted Laser Desorption/Ionization Time of Flight/Time of Flight Mass Spectrometry (MALDI-TOF-TOF MS). A total of 38 gene products were identified from 80 differential spots, and they belong to 5 regulation patterns. Numerous proteins including metabolic enzymes, chaperones and antioxidants were found to be involved in the aging process. These results provide new information that will contribute to our understanding of mechanisms involved in oocyte aging.

## Results

### Analysis of Differentially Expressed Proteins

**Figure 1:**
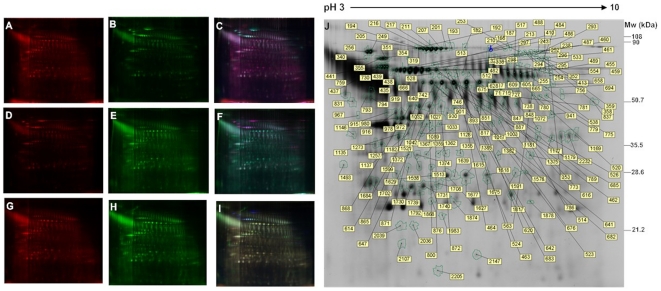
Differentially expressed protein spots shown in 2DE DIGE image. Samples prepared from aging oocytes (**A**, **H**), caffeine treatment (**B**, **D**) and MII oocytes (**E**, **G**) were labeled with Cy3 or Cy5 separately, and images were emerged by software. (**C**, **F**, **I**) the blue spots are internal standard proteins consisting of all samples labeled with Cy2. (**J**) the significantly up-regulated and down-regulated protein spots in the aging group and aging with caffeine group.

Comparison of samples from MII stage oocytes, 24 hours aged oocytes and 24 hours aged and caffeine-treated oocytes is shown in Figure 1A–I. Of 1,334 matched protein spots, 54 were significantly up-regulated or down-regulated in oocytes aged for 24 hours compared to those in the fresh control MII oocytes. As for caffeine treatment, 151 spots were up-regulated or down-regulated compared to oocytes in the MII stage (spots shown in Figure 1J).

### Protein Identification

Differentially expressed spots were digested and analyzed with MALDI-TOF/TOF MS. After searching the pig protein database, 38 protein spots were identified and listed in Dataset S1. Comparison protein levels between normal MII oocytes and aged MII oocytes showed that 23 proteins were up-regulated and 3 proteins were down-regulated. The up-regulated proteins were identified as alcohol dehydrogenase (AKR1A1), aldose reductase (AKR1B1), citrate synthase (CS), GBAK (GNAI3), HLA-B associated transcript 3 (BAT3), adipocyte plasma membrane-associated protein (APMAP), similar to isocitrate dehydrogenase 3 (IDH3B), similar to antioxidant protein isoform 2 (PRDX2), S-adenosylhomocysteine hydrolase (AHCY), trypsin (TRY), UDP glucose pyrophosphorylase (UGP2), annexin A2 (ANXA2), cyclin-dependent kinase 5 (CDK5), beta actin (ACTB), glyceraldehydes-3-phosphate dehydrogenase (GAPDH), hypoxanthine-guanine phosphoribosyltransferase 1 (HPRT1), mitochondrial NAD+isocitrate dehydrogenase 3 beta (IDH3B), mitochondrial solute carrier family 25 member 6 (SLC25A6), nebulin-related anchoring protein (NRAP), proliferating cell nuclear antigen (PCNA), 26 S protease subunit (PSMC), similar to AGAP005293-PA (LOC100153507) and zona pellucida glycoprotein 4 (ZP4). In contrast, beta actin (ACTB), zona pellucida glycoprotein 4 (ZP4), and peptidyl arginine deiminase-like protein (PAD) were identified as down regulated in aged MII oocytes. Among these, the expression level of 4 proteins was significantly changed, namely aldose reductase, mitochondrial solute carrier, antioxidant protein and proliferating cell nuclear antigen.

In caffeine-treated oocytes, 6 proteins were up-regulated and 12 proteins were down-regulated. The up-regulated proteins were DJ-1 protein (PARK7), heat shock 70 kDa protein 1B (HSPA1B), peptidyl arginine deiminase-like protein (PAD), protein glial fibrillary acidic (PGF), similar to GLUD1 protein (GLUD1) and zona pellucida glycoprotein 4 (ZP4). The down-regulated proteins were 90-kDa heat shock protein (HS90A), calreticulin (CALR), heat shock protein 90 kDa beta member 1 (HSP90B1), beta-enolase 3 (ENO3), glyceraldehydephosphate (GAPDH), eukaryotic translation elongation factor 1 alpha 1 (EF1A1), glycoprotein ZP1 (ZP1), gp96/GRP94 (HSP90B1), heat shock 70 kDa protein 5 (HSPA5), peptidyl arginine deiminase-like protein (PAD), zona pellucida 2 glycoprotein (ZP2) and zona pellucida glycoprotein 4 (ZP4). ZP4 have multiple spots in 2D-gel, and the up-regulated and down-regulated ZP4 proteins belonged to different modified products.

In analyzing our results, we found that different spots were identified as the same gene products that also corresponded to different expression levels. This phenomenon may result from protein modification, such as phosphorylation and glycosylation, which was also reported in other 2D-gel studies. For example, we identified heat shock 70 kDa protein 1B corresponding to 2 spots (647, 641) and peptidyl arginine deiminase-like protein corresponding to 4 spots (616, 617, 642, 620) (Figure S1). These spots were distributed either in different molecular masses or in different pH ladders. In addition, proteins of PSMC, ACTB, PAD and ZP4 also showed a similar phenomenon.

### Protein Expression Pattern during the Aging Process

**Figure 2:**
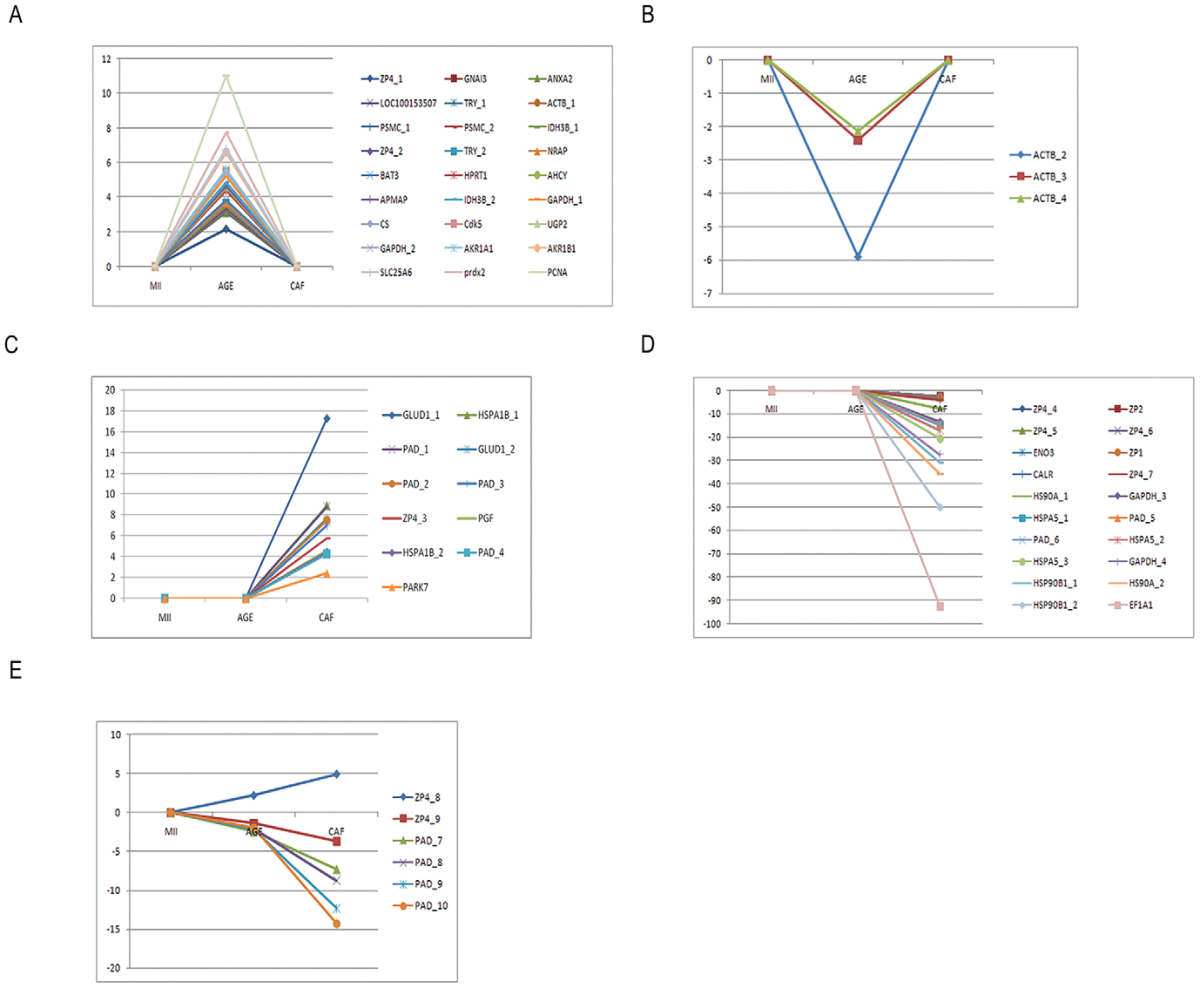
Different protein expression patterns in aging and anti-aging bio-system. The proteins were up-regulated (**A**) or down-regulated (**B**) in oocytes aged for 24 hours, but their expression was restored to the normal level under caffeine stimulation. On the other side, the expression of proteins was not changed in oocytes aging for 24 hours, but was up-regulated (**C**) or down-regulated (**D**) with caffeine stimulation. Some protein expressions were not affected by caffeine (**E**).

Combination of differential results in both aging and caffeine-treated groups showed five patterns based on variations of quantitative protein levels after addition of caffeine (Figure 2). In patterns 1 and 2, the variation of protein levels in the caffeine group was opposite to the 24 hours-aged group (Dataset S2 and S3). In patterns 3 and 4, the gene expression was either increased or decreased by caffeine treatment (Dataset S4 and S5). The differential protein expression was not influenced by caffeine in the latter pattern (Dataset S6). As had been reported in the literature, the aging process in oocytes is delayed by caffeine [15], [16]. The patterns contribute new information on the proteins involved in the aging process and anti-aging mechanisms as important information for treatment. The proteins in patterns 1 and 2 were influenced by oocyte aging and could be reversed by caffeine. As for patterns 3 and 4, it is implied that these proteins play a role in anti-aging or in delaying the aging process.

### Functional Distribution of Differentially Expressed Proteins as Revealed by Gene Ontology

**Figure 3:**
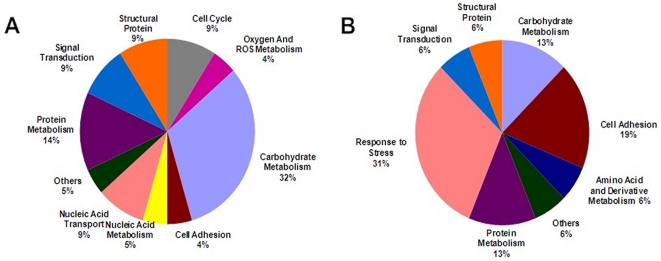
Functional distribution of proteins in the oocytes aged for 24 hours and 24 hours with caffeine. (**A**) functional distribution of proteins involved in oocyte aging for 24 hours. (**B**) functional distribution of proteins involved in oocyte aging with caffeine stimulation for 24 hours.

To investigate the physiological process, we annotated the differentially expressed genes by searching Gene Ontology and performing a literature search. The genes were divided into two categories, the aging-related group and the caffeine-sensitive group; they displayed obvious functional differences (Figure 3). Based on the analysis of our results, we found that different functional gene clusters were expressed in the aging and anti-aging process.

During oocyte aging for 24 hours after maturation, 7 proteins related to carbohydrate metabolism were identified: alcohol dehydrogenase A1, alcohol dehydrogenase B1, adipocyte plasma membrane-associated protein, citrate synthase, glyceraldehydephosphate, isocitrate dehydrogenase 3B and UDP glucose pyrophosphorylase. Among these enzymes, two of them were key enzymes of the Krebs cycle, namely citrate synthase and isocitrate dehydrogenase 3B (Figure S1) and they were up-regulated in the 24 hours-aging group.

With caffeine treatment, we found that about 1/3 of the proteins were involved in the stress response, most of which were heat shock protein family members, including HSPA1B, HSPA5, HS90A and HSP90B1 (Dataset S5 and S6).

### Protein Validation by Western Blot

**Figure 4:**
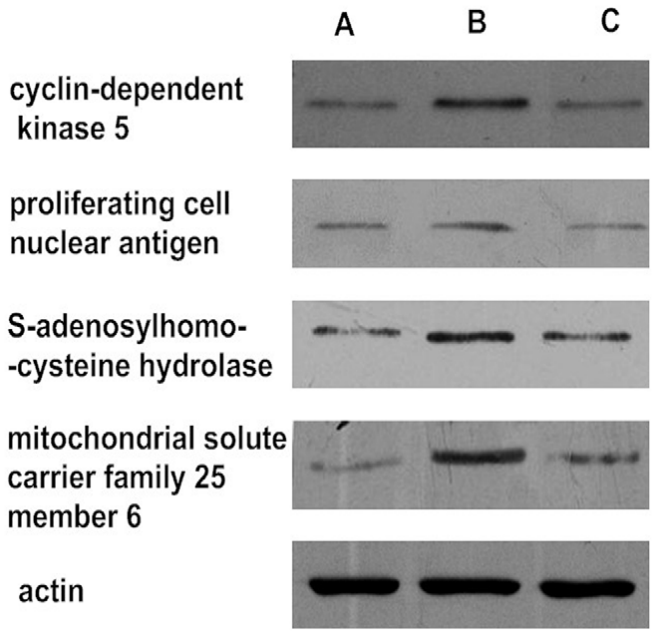
Validation of differentially expressed proteins by Western blot assay. Four proteins were chosen as candidates for validation and the differentially expressed bands were detected in the MII stage (**A**), oocytes aged for 24 hours (**B**) and oocytes aged for 24 hours with caffeine treatment (**C**).

To verify the DIGE results, samples from the MII stage, 24 hours aging and 24 hours aging with caffeine groups were further analyzed by Western blot. We chose proliferating cell nuclear antigen, cyclin-dependent kinase 5, S-adenosylhomocysteine hydrolase and mitochondrial solute carrier family 25 member 6 as candidates in the experiment, whereas alpha-actin was chosen as reference (Figure 4). Our results showed that the genes whose expression was increased in the aging process were reversed after caffeine treatment, showing the same changes as obtained by DIGE analysis.

## Discussion

In most mammals, oocyte meiosis is initiated during fetal development and arrested at the diplotene (germinal vesicle, GV) stage. The quality of oocytes can affect reproduction and embryonic development [1]. Previous studies have characterized aged oocytes on biochemical and molecular biology levels [5], [10], [17], but these studies did not provide an integrative analysis of physiological alterations during the aging process. In the present study, we investigated protein changes of pig oocytes both in oocytes aged after maturation and the response to caffeine treatment. Of the differentially expressed protein spots, 38 gene products were identified and some candidates were validated by Western-Blot experiments.

Our results showed that gene products involved in carbohydrate metabolism, nucleic acid metabolism, protein metabolism, reactive oxygen species and the cell cycle, were expressed differentially. The aging process is reflected in physiological alterations, which provides information for understanding changes during oocyte aging.

In aged heart muscle cells, mitochondrial function was damaged and the Krebs cycle was affected [17]. This phenomenon was also observed in fibroblasts, and it was shown that the respiratory chain became dysfunctional and the activity of citrate synthase decreased [18]. However, these two key enzymes were increased during oocyte aging, which may be considered as compensation to restore mitochondrial function. Moreover, it is suggested that it was compensated by isocitrate dehydrogenase 3 instead of isocitrate dehydrogenase 1 driving the TCA cycle in normal cells. Another up-regulated enzyme in the glycometabolism is glyceraldehydephosphate. It has been shown that the expression of GAPDH was decreased by antioxidant treatments in aged neural cells from Alzheimer's disease patients [19]. This result was also obtained in our data; by adding caffeine, the expression level of GAPDH was restored and it was similar to that in non-aged oocytes. As for other enzymes in the carbohydrate metabolism, there was no evidence to prove their correlation to aging.

Cell cycle related proteins play important roles in cell proliferation and cell division. Cyclin dependent kinase 5 (CDK5) and proliferating cell nuclear antigen (PCNA) were both up-regulated in our results. CDK5 is a serine-threonine kinase and expressed in the nerve systems. It can interact with G1 cyclin and phophorylate H1, tau and MAP2 [20]. A previous study showed that a series of cyclins including CDK5 were up-regulated in aged nerve cells [21]. PCNA is a protein with multiple functions and it is used as cell cycle marker protein important for DNA replication and repair [22]. In clinical studies, it was discovered that the concentration of PCNA was positively correlated with age by assaying PCNA expression in persons of different ages. Our study confirmed the differential expression of CDK5 and PCNA, as reported in the literature.

It has been well documented that oxidative stress occurs during cellular aging and is associated with the production of free radicals and reactive oxygen species [23], [24]. Hence, anti-oxidative mechanisms are critical for cell survival. Based on our data, an anti-oxidative protein, peroxiredoxin 2 (PRDX2), was up-regulated. It is the third most abundant protein in the erythrocyte and considered to play a major role in the cell's oxidative defenses [25]. Moreover, a clinical survey showed that the expression of PRDX2 was up-regulated with increasing age [26], which implied a protective effect of PRDX and resistance to aging.

As indicated above, physiological aging can be prolonged by caffeine and most of the proteins' expression was restored to the normal level after caffeine treatment, as shown in our data. However, as shown in Figure 3B, numerous proteins became changed when caffeine was added. These proteins might be effective on molecular levels to participate in anti-aging mechanisms.

Our data show that HSPA1B was increased after caffeine treatment, but HSPA5 was down-regulated. According to a previous study, expression of HSP 70 was decreased in aged mouse brain and could be reversed by estrogen [27]. There was also an age-related decrease in HSP70 in human serum and lymphocytes [28]. As for HSP90, all isoforms were down-regulated under caffeine stimulation. A previous study examining HSP levels found that HSP90 was positively correlated to aging [29]. Our data showed the opposite pattern, which suggests differential regulation in different cells and tissues.

Many kinds of protein modifications exist, such as phosphorylation, glycosylation and acetylation, which can change a protein's molecular weight or isoelectric point. One advantage of the 2DE method is the ability to display these protein changes. Here, we found that some of the proteins were distributed in a series of spots, including HSP90, ZP1, PAD and GAPHD (Figure S1). The *pI* shift of these proteins may have resulted from phosphorylation. Peptidylarginine deiminases (PAD) are a group of posttranslational modification enzymes that citrullinate protein arginine residues. Studies on aging persons indicated that this enzyme may play a role in the initiation and/or progression of Alzheimer's disease by citrullinating other proteins [30]. Phosphorylation of this enzyme in cell aging was not reported. GAPDH is a key enzyme in glycolysis that catalyzes the first step in the pathway by converting D-glyceraldehyde 3-phosphate (G3P) into 3-phospho-D-glyceroyl phosphate. With proteomic analysis, many proteins including GAPDH were significantly reduced in brain of an aged dog model [19]. Although the decrease of these proteins was reported in other tissues, the modification was first discovered in our study, which provides new information for understanding the involvement of these proteins involved in aging. Whether they are phosphorylated remains to be determined in future studies.

In conclusion, a powerful proteomics tool has been employed to analyze the protein profile changes during pig oocyte aging and effects of caffeine on these changes. With 2D DIGE and MALDI-TOF/TOF MS, we identified down-regulated and up-regulated proteins in aged oocytes and in caffeine-treated oocytes. Some interesting proteins were also verified by Western-blot assay, which may lead to further studies on the regulation of oocyte ageing.

## Materials and Methods

### Ethics Statement

Porcine ovaries used in this study were obtained from a local slaughterhouse named Third Meat Processing Factory in Beijing, P.R. China and all procedures were conducted in accordance with policies promulgated by the Ethics Committee of the Institute of Zoology, Chinese Academy of Sciences.

### Reagents

All chemicals used in this study were purchased from Sigma Chemical Company (St. Louis, MO), unless otherwise noted. TCM119 cell culture medium was purchased from Gibco Carlsbad, CA). Fetal bovine serum was purchased from Invitrogen. Cy2, Cy3, and Cy5 were purchased from GE Healthcare. Dimethylformamide was purchased from Aldrich. DTT, urea, agarose, glycerol, bromphenol blue, CHAPS, mineral oil, acrylamide, Bis, Tris base, glycine, SDS, iodoacetamide, ammonium persulfate, TEMED, Immobiline DryStrip gels (24 cm, pH 3–10), and Bio-Lyte solutions (pH 3–10) were purchased from Bio-Rad. Thiourea was purchased from Fluka (Buchs, Switzerland). Protease inhibitor mixture was purchased from Roche Applied Science. ACN and methanol were purchased from Fisher. TFA was purchased from Merck. Trypsin (sequencing grade) was purchased from Promega (Madison, WI). All buffers were prepared with Milli-Q water (Millipore, Bedford, MA).

### Oocyte Collection and *In Vitro* Maturation

Porcine ovaries were obtained from prepubertal gilts at a local slaughterhouse and transported to the laboratory within 1 hour. The ovaries were maintained in 0.9% NaCl solution containing penicillin G (75 mg/mL) and streptomycin sulphate (50 mg/mL) at 34°C∼36°C. After washing in physiological saline 3–5 times, cumulus-oocyte complexes (COCs) were aspirated from antral follicles (3–6 mm in diameter) with an 18-gauge needle fixed to a 20 ml disposable syringe. After three rinses in washing medium (TCM-199 medium supplemented with 2.2% NaHCO_3_), COCs with uniform cytoplasm and a compact cumulus mass were selected for the experiments.

*In vitro* maturation was conducted as reported previously [12]. Briefly, oocytes were cultured in TCM199 supplemented with 75 mg/L penicillin and 50 mg/L streptomycin, 0.57 mM cysteine, 0.5 mg/ml FSH, 0.5 mg/ml LH, and 10 ng/ml EGF. Approximately 90 to 100 COCs were cultured in 1 ml maturation medium in a 12-well dish, which was covered with liquid paraffin oil for up to 44 h at 39°C in an atmosphere of 5% CO_2_ in air and saturated humidity. After maturation culture for 44 h, some of the oocytes were collected for analysis, while the remaining oocytes were further cultured for an additional 24 hours with or without 5 mM caffeine (Sigma), resulting in a total maturation culture time of 68 hours.

### Sample Preparation, 2D-DIGE and Image Analysis

The protein profile analysis was commercially conducted by the Dept of Genomics and Proteomics, Beijing Institute of Radiation Medicine following the protocol published previously by Sun *et al.* [31], and adapted as described below. In each group, more than 3000 oocytes were disrupted by applying ultrasonic wave and proteins were extracted by using lysis buffer (consisting with 7 M urea, 2 M thiourea, 4% CHAPS, 65 mM dithiothreitol, and 1% protease inhibitor cocktail). Protein extract concentration was determined by the BCA method and pH was adjusted to 8.5 with 50 mM NaOH. Next, the protein concentration was adjusted to 5 mg/ml with lysis buffer. Equal amounts of proteins from 3 samples were pooled together as internal standard. Proteins from MII oocytes, aging oocytes and aging oocytes treated with caffeine were randomly labeled with Cy3 or Cy5, whereas internal standards were labeled with Cy2 using 400 pmol of fluorochrome/50 g of protein. Labeling was performed for 30 minutes on ice in the dark.

Reactions were then stopped by addition of 1 µl lysine (10 mM) for 10 min on ice in the dark. Fifty-microgram Cy3- and Cy5-labeled samples from each group were combined before mixing with 50 µg of Cy2-labeled internal standard. Next, an equal volume of 2 µl sample buffer (7 M urea, 2 M thiourea, 4% CHAPS, 1% Bio-Lyte, pH 3–10, 20 mg/ml DTT) was added to the sample, and the total volume was made up to 450 µl with rehydration buffer (7 M urea, 2 M thiourea, 4% CHAPS, 0.5% Bio-Lyte, 10 mg/ml DTT).

Samples were actively rehydrated into 24-cm pH 3–10 IPG strips (Bio-Rad) at 17°C for 7 h using a Protean IEF cell (Bio-Rad). Isoelectric focusing was performed for a total of 80 kV-h, involving desalting (250 V in 30 min), boosting voltage (held at 1,000 V for 1 h, ramped to 10,000 V in 5 h) and holding (10,000 V for 70 kV-h). The IPG strips were equilibrated in equilibration buffer (6 M urea, 2% SDS, 50 mM Tris-HCl, pH 8.8, 30% glycerol) supplemented with 0.5% DTT for 15 min at room temperature followed by 4.5% iodoacetamide in equilibration buffer for another 15-min incubation at room temperature.

IPG strips were placed on top of 12% homogeneous polyacrylamide gels that had been precast in low fluorescence glass plates using an Ettan DALT twelve gel caster. The second dimension SDS-PAGE was carried out using the Protean Plus system (Bio-Rad). After 2DE, gels were scanned on the Typhoon 9410 scanner with Ettan DALT gel alignment guides using excitation/emission wavelengths specific for Cy2 (488/520 nm), Cy3 (532/580 nm), and Cy5 (633/670 nm). The intensity was adjusted to ensure that the maximum volume of each image was within 60,000–90,000. Experiments were repeated three times.

### Data Analysis

Analysis of 2D DIGE was performed using DeCyder 5.02 software (GE Healthcare) according to the manufacturer's suggestion. Briefly, the DeCyder biological variation analysis module was used to detect spots and simultaneously match all 204 protein spot maps from 3 gels. All matches were also confirmed manually. The paired t test was used for statistical analysis of the data. Different density of protein spots for the MII stage, 24 hours-aged and 24 hours-aged with caffeine groups were marked. Only spots with variations more than 1.2 were selected and identified. Spot picking and in-gel digestion was carried out with preparative gels. Two-dimensional electrophoresis was performed as described under “2D DIGE and Imaging” except that the IPG strips were loaded with 800 µg of protein, and gels were stained with Coomassie Brilliant Blue. Protein spots of interest were excised and destained with 25 mM ammonium bicarbonate in 50% ACN. Gels were then completely dried by centrifugal lyophilization. In-gel digestion was performed with 0.01 µg/µl trypsin Promega (Madison, WI) in 25 mM ammonium bicarbonate for 15 h at 37°C. The supernatants were collected, and the tryptic peptides were extracted from the gel sequentially with 5% TFA at 40°C for 1 h and with 2.5% TFA, 50% ACN at 30°C for 1 h. The extracts were pooled and completely dried by centrifugal lyophilization.

### Protein Identification

Peptide mixtures were dissolved in 0.5% TFA, and 1 µl of peptide solution was mixed with 1 µl of matrix (4-hydroxy-α-cyanocinnamic acid in 30% ACN, 0.1% TFA) before spotting on the target plate. PMF and sequence analysis were carried out on a MALDI-TOF-TOF MS (4800 Proteomics Analyzer, Applied Biosystems). Peptide mass maps were acquired in positive reflection mode, averaging 1500 laser shots per MALDI-TOF spectrum and 3000 shots per TOF/TOF spectrum (the resolution was 20,000). The 4800 calibration mixtures (Applied Biosystems) were used to calibrate the spectrum to a mass tolerance within 0.1 Da. Parent mass peaks with a mass range of 600–4,000 Da and minimum signal to noise ratio of 15 were picked out for tandem TOF/TOF analysis. Combined mass and mass/mass spectra were used to interrogate sequences in the NCBI pig protein database with the MASCOT database search algorithms (version 1.9). In the searching parameter, modification was set as carbamidomethylation, oxidation, and a maximum of one missed trypsin cleavage was permitted. Tolerance of precursor and fragment ions were both set to 0.2 Da. All of the automatic data analysis and database searching were fulfilled by the GPS Explorer™ software (version 3.6, Applied Biosystems). Contaminant proteins, such as keratin (from skin or hair), were excluded manually. The confident identification had a statistically significant (p≤0.05) protein score (based on combined mass and mass/mass spectra) and best ion score (based on mass/mass spectra). Redundancy of proteins that appeared in the database under different names and accession numbers was eliminated. If more than one protein was identified in one spot, the single protein member with the highest protein score (top rank) was singled out from the protein family. The molecular weight and pI value of most proteins were consistent with the gel regions from which the spots were excised.

### Western Blot

Proteins from 3 groups were separated on 12% polyacrylamide gels and transferred to PVDF membranes (Amersham Biosciences,Piscataway, NJ). These blots were incubated for 2 h at room temperature in Tris-buffered-saline with Tween (20 mM Tris-Cl, 140 mM NaCl, pH 7.5, 0.05% Tween 20) containing 5% skim milk. Primary antibodies used were anti-proliferating cell nuclear antigen monoclonal antibody (diluted 1∶500, Sigma), anti-cyclin-dependent kinase 5 (diluted 1∶1000 Sigma), anti-S adenosylhomocysteine hydrolase (diluted 1∶1000 Promega) and anti-mitochondrial solute carrier family 25 member 6 (diluted 1∶200 Sigma). Alpha-actin was selected as internal reference, and the monoclonal antibody was diluted into 1∶1000 (Sigma). Blots were incubated with primary antibodies for 2 h at room temperature. After washing three times in Tris-buffered-saline with Tween, blots were incubated with horseradish peroxidase-conjugated secondary antibody (diluted 1∶10,000, Santa Cruz Biotechnology) for 1 h at room temperature. Immunoreactive complexes were visualized using ECL reagents (Santa Cruz Biotechnology).

## Supporting Information

Figure S1 **Multiple spots corresponding to one protein.** In 2D-gel, one gene product could be identified from several spots. Generally, these spots have different *pI*, which may be modified by phosphorylation, such as HSP90, ZP1, PAD and GAPDH.
(TIF)

Dataset S1 **The information of total identified proteins after database searching.**
(XLS)

Dataset S2 **The variation of protein levels in pattern 1.** The number in column of “aging group” represents ratio of up-regulated proteins in aged oocytes comparing to caffeine treatment.
(XLS)

Dataset S3 **The variation of protein levels in pattern 2.** The number in column of “aging group” represents ratio of down-regulated proteins in aged oocytes comparing to caffeine treatment.
(XLS)

Dataset S4 **The variation of protein levels in pattern 4.** The number in column of “caffeine treatment group” represents ratio of up-regulated proteins in caffeine treatment comparing to aged oocytes.
(XLS)

Dataset S5 **The variation of protein levels in pattern 5.** The number in column of “caffeine treatment group” represents ratio of down-regulated proteins in caffeine treatment comparing to aged oocytes.
(XLS)

Dataset S6 **The differential protein expression was not influenced by caffeine treatment.**
(XLS)
