# Supplementary figures and images for: Protein Profile Changes during Porcine Oocyte Aging and Effects of Caffeine on Protein Expression Patterns
Source: PLoS One. 2011 Dec 16;6(12):e28996. doi: 10.1371/journal.pone.0028996 (PMC3241687; doi:10.1371/journal.pone.0028996)

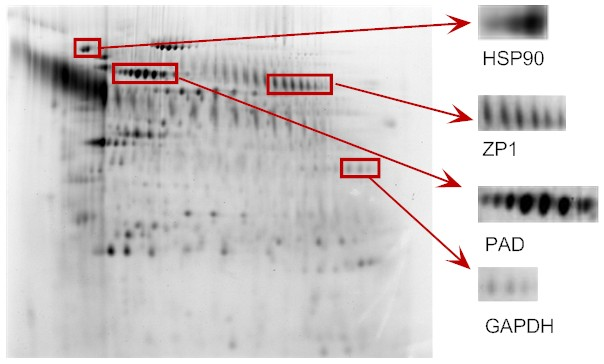

Supplement: Figure S1 — Multiple spots corresponding to one protein. In 2D-gel, one gene product could be identified from several spots. Generally, these spots have different pI, which may be modified by phosphorylation, such as HSP90, ZP1, PAD and GAPDH. (TIF) [file pone.0028996.s001.tif]
